# Health security—Why is ‘public health’ not enough?

**DOI:** 10.1186/s41256-024-00394-7

**Published:** 2025-01-03

**Authors:** Delaram Akhavein, Meru Sheel, Seye Abimbola

**Affiliations:** Faculty of Medicine and Health, Sydney School of Public Health, The University of Sydney, Sydney, NSW Australia

**Keywords:** Health security, Global health policies, Global health, Health securitisation, Infectious diseases, Power dynamics

## Abstract

There is a growing tendency in global discourse to describe a health issue as a security issue. But why is this health security language and framing necessary during times of crisis? Why is the term “health security” used when perhaps simply saying “public health” would do? As reference to ‘health security’ grows in contemporary discourse, research, advocacy, and policymaking, its prominence is perhaps most consequential in public health. Existing power dynamics in global health are produced and maintained through political processes. Securitisation of health, which facilitates urgent and exceptional measures in response to an event, is a politically charged process with the tendency to further marginalise already marginalised individuals, groups, and nations. By exploring the ethical and practical consequences of a powerful actor’s move to securitise health, the essay highlights the importance of considering the perspectives and well-being of marginalised individuals, groups and nations who may be impacted by the move. The essay challenges the assumption that securitising health or framing health as a security issue necessarily leads to good outcomes. It highlights the historical roots and explores the contemporary implications of “health security”, and invites critically informed discourse on its use within global health.

## Introduction

Our world is shaped by a complex history of tension [1, 2]. This history is marked by the production and maintenance of power and hierarchy through the inclusion and simultaneous exclusion of certain groups of people and places [2]. This dynamic is evident in International Health,^1^ which originated in the internationally standardised quarantine regulations set up during the International Sanitary Conferences initiated by France in 1851. International Health was shaped through the colonial encounter—the need to protect the “metropole” from the periphery—as these International Sanitary Conferences sought to safeguard Europe from the perceived threat posed by “exotic” diseases [3, 4]. Such tendencies continue to inform how international health policies (most notably those related to infectious diseases) are developed and disseminated—enabling modern sovereign powers to maximise their power and control [5]. It is not by accident that the way certain health issues are prioritised and operationalised and agendas are developed is tightly bound to those whose humanity and interests are the core concerns of those with the power to decide. These motivations can result in poorer health outcomes and a diminished emphasis on addressing the concerns of individuals, groups, and nations with *less* power to decide.

As the health security discourse in the field of global (public) health^2^ continues to grow, making “health security” nearly synonymous with “global health”, much of the existing (critical) scholarship on how health is framed within the security context and its impact on global governance, policies, and international relations has primarily emerged from disciplines such as International Relations [6, 7] and Security Studies [8, 9]—a scholarship that global health practitioners may not readily engage with, especially given the policy- and practice-oriented nature of the field. However, it is crucial to engage and understand how such framing has evolved and how it functions in public health [10], with varying outcomes for different individuals, groups, and nations. Therefore, this reflective essay, aimed at global health audiences directly involved with policy and practice, is written to encourage critical engagement with an examination of the political salience of health security, promote discussions that question its necessity, and consider the tangible implications of its use in different contexts. By doing so, the essay seeks to connect theoretical discussions with practical applications and realities so that the language and framing of health security are critically assessed when applied to global health initiatives and policies.

## Framing health as ‘security’

Why is the language or framing of health security necessary in the first place? Why – or perhaps when – are we prompted to go beyond simply characterising a health issue as a public health issue?

**Fig. 1:**
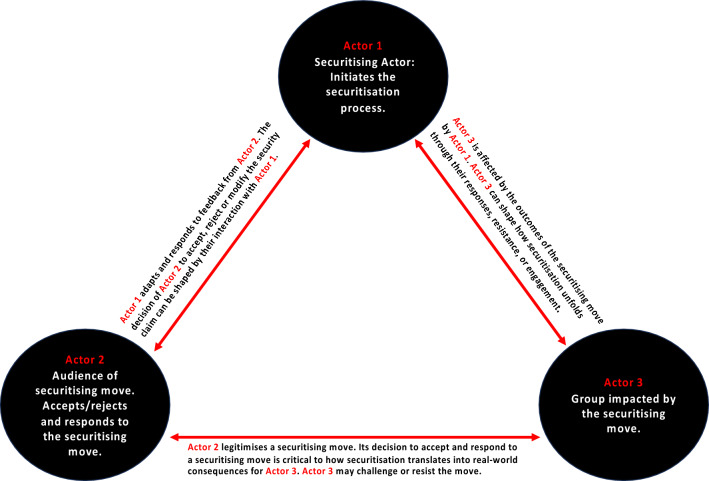
The Actor 1-Actor 2-Actor 3 relationship explaining their securitisation roles and experiences

Health has never been *un-*strategic [4]. Securitisation, based on the Copenhagen School’s Securitisation framework, typically involves two main actors: one initiating the speech act (actor 1) by sounding the alarm on security concerns and another who must accept and respond to this alert (actor 2) [8]. However, the framework does not explicitly consider a third group of actors—those potentially affected by a securitising move or those at the receiving end of the securitising move (actor 3). In our conceptualisation of health securitisation (Fig. 1), we depart from the traditional Securitisation Framework [8] by explicitly taking into consideration the roles and experiences of actor 3, thus opening up space to theorise this often-neglected group of health securitisation actors (in the form of individuals, groups or nations) and (re)surface the need to interrogate more closely the potential motivations and consequences of the health security framing.

Actor 1, the securitising actor (e.g. an international organisation), can raise concerns and frame health issues (e.g. infectious disease outbreak) as security threats. By doing so, actor 1 makes claims for ‘extraordinary measures’, claims that justify heightened responses, often aimed at influencing policy, resource allocation, and collective action. Actor 1 aims to elevate the perceived threat above normal political processes by initiating the securitisation process, creating a sense of urgency and prioritisation within global and national frameworks. Actor 2, the audience (e.g. governments of other countries), plays a critical role by validating or rejecting these security claims. Often, actor 2 determines whether the securitised framing of the issue gains traction. The relationship between actor 1 and actor 2 is not static. It is shaped by their proximity and interaction, including shared interests, political influence, relative power, and social positioning. Furthermore, actor 2’s stance can be shaped by their proximity and relationship to actor 3, whether geographically, socially, or politically—there may be a stronger inclination to prioritise actor 3’s experiences depending on how well actor 2 is connected to actor 3. Actor 3 can also resist or challenge either actor 1 or actor 2’s role and influence. In our conceptualisation, actor 3, the affected individuals, groups, or nations, experience the consequences of a securitisation process. Their realities are shaped by the outcomes, making their well-being a central yet often overlooked element in the broader discourse. While they are typically not active participants in decision-making, they are not entirely without agency. Actor 3 can shape how securitisation unfolds through their response, resistance, or engagement. Together, the evolving interactions shaped by power relations between actor 1, actor 2, and actor 3 in a setting influence how health is framed and acted upon in global or national policy.

Therefore, it is important to consider the notion that health securitisation is perhaps not something that happens to disease [4] but to individuals, groups or nations. Consider, for example, the historical utilisation of health as a tool by Western imperial powers used health as a tool for the purpose of colonisation [3, 11–13]. That is, how colonisers (actor 1) appealed to their military (actor 2) to secure the health of the colonisers (actor 1). However, colonisers also sought to secure the health of colonised populations (actor 3), not for their own sake, but to ensure the effective extraction of resources and manpower. [4, 14–16]. The colonised populations bore the brunt of these strategies. While the focus is usually on the dynamic between the alerting (actor 1) and reacting actors (actor 2), the impact on the third group of actors (actor 3) should be central to any analysis of health security.

## Contemporary discourse

The explicit framing of health as a security issue is often linked to the early 2000s —conflated by 9/11- when the UN Security Council adopted resolution 1308, portraying HIV/AIDS as a security issue motivated by the inherent concern around combat effectiveness. [14, 32]. Ethical implications of framing HIV/AIDS as a matter of security have been challenged and explored [15, 18]. Nonetheless, such framing has produced policy responses for the protection of high-income individuals, groups, or nations, as evidenced by a focus predominantly overseas [30, 32, 33]. While this does not negate that the funding and resources it has leveraged have benefited recipient countries, it does raise a critical question: when—or why—is it not enough to simply call a health issue that requires attention, even urgent attention, a public health issue? Might there be a moral dilemma in pursuing a security-charged agenda?

The United States Centre for Disease Control and Prevention (CDC) defines health security as a state of resilience specifically to infectious disease threats; i.e., health security as “resilient public health systems that can prevent, detect, and respond to infectious disease threats, wherever they occur in the world” [28]. Note that in this definition, health security describes the status of public health systems—i.e., resilience to specific threats. Given the United States’ leadership in health security agenda setting, the CDC’s conceptualisation of health security shapes and influences how public health emergencies are conceptualised (under health security framing) and addressed globally. The World Health Organisation’s (WHO) definition, although more dynamic, presents health security as “…activities required, both proactive and reactive, to minimise the danger and impact of acute public health events that endanger people’s health across geographical regions and international boundaries” [29]. However, WHO’s definition is still threat-focused—what is being secured against are acute public health events. This securitised language is echoed in the commitments made under the International Health Regulations 2005 IHR (2005) [30]. Countries are encouraged to develop the capacity to “detect, assess, report, and respond to potential public health emergencies of international concern” [31]. While such definitions have elements of “protectionism” that create a cloak of benevolence, it is important to understand who and what is being protected and the motivations behind how certain tools can be deployed to different ends.

Nonetheless, there continues to be a shift towards framing health issues through a security lens [18]. It has been argued that this is because of high-income countries acting in their own self-interest [7, 34], which is seen in response to infectious disease outbreaks such as Ebola [18], Zika [19] and most recently, COVID-19 [20] because what constitutes a health security threat and who wields power to label it as such remains contentious [21–23]—just as in the nineteenth century, International Sanitary Conferences were contentious in terms of which (European) countries had the power to decide the fate of others [24]. At its core, the language of security creates a sense of urgency and exceptionalism [8]; however, what constitutes a threat against an individual, group, or nation is less dependent on whether the security threat actually exists but on the type of health issue and whether the political/securitising actors can successfully and effectively frame the health issue as a ‘threat’ to a potentially susceptible or welcoming audience—’’It is a choice to phrase things in security… terms, not an objective feature of the issue’’ [8, 25]. When successful and effective, a securitising move is made such that it results in mobilising multilateral/multisectoral responses and resources outside the domain of everyday politics—or “normal politics”—with the (implicit) goal of safeguarding the interests of powerful individuals, groups or nations [9, 26].

The 2014 Ebola outbreak in West Africa, which occurred during the development of the Global Health Security Agenda (GHSA), has also provided fertile ground for heightened global interest in the use of health security language and framing in the last decade—once Ebola’s potential for spread beyond its original ‘tri-border’ region was recognised internationally [34]. The everyday citizens of Guinea, Liberia, and Sierra Leone (actor 3) were not only the primary victims of the outbreak but also very much at the receiving end of *securitised* public health efforts in the form of coercion and containment [18] that primarily aligned itself with national security forces at the hand of its governments (actor 2)—endorsed and promoted, in part by governments of certain high-income countries and global organisations (actor 1), aligning with their own agendas and priorities. The United States (US) and the United Kingdom (UK), which were actively involved in the response, in many ways, have been at the forefront of championing practices that stem from framing health issues as security threats. This commitment is also evident in actions such as the creation of the Bureau of Global Health Security and Diplomacy by the US State Department in 2023—the latest move in its health security development timeline^3^ [27]. The UK’s transformation of Public Health England into the UK Health Security Agency during the COVID-19 pandemic also nudges towards a shift and normalisation of health security framing. While such developments gather pace, there has been limited critique globally in the public health policy and practice beyond what health security means and does theoretically [4] compared to its practical impact on public health systems in different contexts, specifically in relation to individuals, groups or nations (actor 3).

The covert securitisation of health events through the declaration of Public Health Emergencies of International Concerns (PHEICs)—framing pandemics in terms of security, triggering extraordinary measures such as travel restrictions, quarantine, and large-scale mobilisation of resources—has been a driving force behind increased investment in pandemic preparedness and response activities [32, 35]. Indeed, such investments are needed. However, it is equally essential to recognise the nuanced motivations underlying such financial mobilisation. At the international level, this framing has been aligned with a tendency to prioritise national agendas over comprehensive global public health responses. This is especially the case when outbreaks extend beyond the border of their origin, most notably the 2014 West African Ebola outbreak [6, 23, 26, 32, 36], influencing how future ‘threats’ are perceived and managed. Since securitisation, an inherently undemocratic process, is often triggered in the immediate environment of a health emergency, its context-specific, longer-term impacts, especially on actor 3, are not readily considered. The urgency and exceptionalism inherent in the health security language and framing can reduce policies and practices to mere biomedical solutions rather than a deeper understanding of the political, economic and social factors intrinsic to strengthening health systems.

The perception and reality that the language and framing of health security prioritise preventing threats to Western countries, while the rest of the world is often viewed as the source of threat [37, 38], is deeply rooted in the legacy of colonisation and is well recognised [36, 39–41]. These patterns have persisted into the present, where former colonial powers can, through the influence of soft power, influence global narratives and priorities reinforced by their economic dominance [1, 3]. One manifestation of the awareness of such logic can be seen in Indonesia’s response to the 2006 H5N1 pandemic. Indonesia refused to share its national virus samples with the international community due to concerns about a lack of assurance of the mutual benefits of sharing samples and data, such as vaccine access [42, 43]. This refusal to share viral samples exacerbated political tensions and undermined the perceived benefits of health security—international cooperation. Such a response by Indonesia indicates the acknowledgment of the inequalities within global health governance and resistance to the inherent power imbalances within such structures [42], which manifest in different securitising moves. Sample and data sharing can no longer be viewed as merely scientific and technical in a space that has historically bred power imbalances and “uneven geographic distribution of biomedicine” [42].

Narratives such as these have continued to persist. Most recently, South Africa demonstrated high detection and surveillance capacity by sharing a new SARS-CoV-2 Omicron Strain in November 2021 [43]. However, governments of Western countries (actor 1) responded with a somewhat colonial logic directed at their population (actor 2) with dire consequences for Southern Africans (actor 3). Despite the absence of scientific evidence of the risk posed by the detection, South Africa and nearby countries were met with travel bans [44]. By this time, it was scientifically clear that detection no longer meant origin, and by the time a strain was detected, it had likely spread outside of its immediate origin [44, 45]. Nevertheless, South Africa was penalised despite demonstrating the exact capacities and capabilities advocated by the IHR for health security [32]. Scholars such as Silva and Smith have since advocated a more conditionally reciprocal approach to data sharing and collaboration during emergencies (health security events) that extends beyond symbolic expressions of solidarity – one with guaranteed equitable benefits for all countries regardless of their geographic location or economic status [43].

Ultimately, what matters is considering who is most affected by health security framing (actor 3). There is a need, for example, to consider the divide between the intended global strategies [46] and how such language and framing can unravel as action in different contexts—especially for less powerful individuals, groups, and nations (actor 3). The imperial echo of “front liners fighting” the outbreak with “targeted” responses in many ways shaped how some countries responded to COVID-19, heightening insecurities within communities by allowing the mobilisation of ‘exceptional’ governmental powers [47]. In Uganda, President Museveni’s perceived success in maintaining low COVID-19 case numbers, despite the local impacts due to the enforcement of military action and centralised authority, earned him recognition for extraordinary leadership by the British Medical Journal [48, 49]. Similarly, in the Philippines, President Duterte’s “war on drugs” was extended as a “war against COVID-19” [50], with repressive measures coming at a painful cost to communities with coercive measures [51]. Australia, often seen as more democratic, placed 3000 residents, a significant proportion from non-European backgrounds, in inner-Melbourne public housing towers under home detention [52], where members of the community found themselves without food, medicine and everyday essentials. The framing of health as a security issue supported this selective over-policing of certain communities, showcasing how securitised responses can lead to different outcomes for different groups, even within one country. What remains common across these examples is the group most impacted (actor 3)—already marginalised populations.

## Future considerations

Despite existing research, insight, and evidence on the outcomes of securitised responses, there are ongoing efforts to broaden its reach, that is, to frame other health issues, such as non-communicable diseases, maternal health, and climate change, within the context of health security [53–56]. This securitising move is being enacted to leverage the perceived benefits of health security framing, namely, to garner political support, increase funding, and mobilise resources. The power dynamics inherent in securitising such issues raise questions about whose priorities matter and who possesses the influence to utilise this approach effectively. The success of fully securitising these issues remains limited, and the prospects of reaping the potential benefits now or in the future remain uncertain.

The enthusiasm for these efforts ought to be tempered by the experience that securitising an issue does not necessarily translate to greater attention or funding. Consider, for example, that despite mounting evidence linking climate change to an increased risk of epidemic diseases and poor health outcomes [57], governments such as the UK have failed to show meaningful commitment to addressing the climate crisis—at the least, as part of bigger efforts to ‘prevent and respond’ to infectious disease. Perhaps it is easier to imagine the UK government securitising its borders against displaced communities due to climate change than taking action that may hurt its economy in the short term but contribute to mitigating climate change. This is a pattern of response exemplified by the UK government’s decision in 2023 to grant over 100 new drilling licenses in the North Sea [58]. This highlights how political and economic factors shape perceptions of what constitutes a genuine ‘health security threat’ and whose priorities drive governmental (in)action.

There are also growing calls for the deepening and ‘reimagining’ of health system strengthening in terms of health security [59], that is, for example, consider prioritising or making the case to prioritise Universal Health Coverage (UHC), Primary Health Care (PHC), and One Health given their supposed potential to contribute to achieving health security [60]. It argues that if these approaches to improving health become integrated into the ‘health security architecture’, it would reorient our understanding and operationalising of health security to adequately address and consider the social, economic, political, and ecological factors influencing health [56, 59, 61–63]. While this may sound like a more logical and ‘sustainable’ approach, it reinforces the legitimacy of the health security framing [64], further securing its position within public health without critically assessing and questioning whose vested interests are at the forefront of such framing. In reframing things that are desirable in terms of health security, we may lose our ability to advocate for them simply in terms of their contribution to public health or health equity.

The impact of using security language on our ability to explore, analyse, and comprehend complexity in relation to health is significant. As an interpretive device, the language and framing of ‘health security’ can help bring certain public health issues into sharper relief; however, doing so also reinforces existing power dynamics and hierarchies inherent in the concept while also obscuring other issues from attention. It is crucial to understand and examine how such language and framing affect the less powerful or marginalised (actor 3). We should also question what it does to the respondent’s (actor 2) ability to (mis) understand the world, while self-interest-driven motivations are endorsed by a powerful few (actor 1).

Health security’s historical origins, intertwined with neo-colonial undertones, highlight the complex narrative that continues to shape its path [17, 33]. Perhaps we do not need to navigate the delicate balance between framing health issues as security threats and how best to incorporate the concept into the broader health system, but rather question, “Why is public health not enough?”. When a health issue is securitised, can it revert to a state of normalcy? Or, when the governance system reorganises itself to treat a health issue as a security issue, does it ever return to a *de*securitised state? [4] The history of public health as a field is marked by an accumulation of post-securitisation changes from its colonial entanglements till today. If we are participating in health discussions using the health security framing and endorsing its use by the language we use, then we have a responsibility to confront and engage ethically with its complexities, its origins, its meanings, and its implications on *all* individuals, groups and nations.

## Data Availability

Not applicable.

## Footnotes

1 The term ‘International health’ is used here in line with the period that this part of the essay describes and reflects upon; that is, where international health originated from in the 1850s.

2 The piece replaces ‘International health’ with ‘Global health’ or ‘Global public health’ to reflect the evolution of the field over time and the contemporary labels of the field that was historically referred to as ‘International Health.’.

3 Formation of the Global Health Security Initiative (2001), The National Strategy for Countering Biological Threats and the National Health Security Strategy (2009, Global Health Security Strategy (2015), Global Health Security Strategy (2019).
